#  Terbinafin 1% Cream and Ketoconazole 2% Cream in the Treatment of Pityriasis Versicolor: A randomized comparative clinical trial

**DOI:** 10.12669/pjms.306.5509

**Published:** 2014

**Authors:** Farrokh Rad, Bahram Nik-Khoo, Roxana Yaghmaee, Fardin Gharibi

**Affiliations:** 1 Farrokh Rad, MD, Associate Professor of Dermatology. Kurdistan University of Medical Sciences, Sanandaj, Iran; 2 Bahram Nik-Khoo, Associate Professor of Pathology, Medical Faculty, Kurdistan University of Medical Sciences, Sanandaj, Iran; 3 Roxana Yaghmaee, MD. Assistant Professor of Dermatology, Kurdistan University of Medical Sciences, Sanandaj, Iran; 4 Fardin Gharibi, MsPH Health Management, Deputy of Research and Technology, Kurdistan University of Medical Sciences, Sanandaj, Iran

**Keywords:** Terbinafine cream, Ketoconazole cream, Pityriasis versicolor, Cure rate

## Abstract

***Objective:*** To make a comparison between terbinafine 1% cream and ketoconazole 2% cream in the treatment of pityriasis versicolor.

***Methods:*** This randomized single blind study included 110 patients with clinical diagnosis of pityriasis versicolor and positive mycological test for Malassezia furfur. The patients were randomly assigned to two groups. Group 1 used terbinafine cream and group 2 applied ketoconazole cream on the skin lesions for two weeks. Each group consisted of 55 patients. Clinical and mycological examinations were performed at baseline, at the end of the 2^nd^, 4^th^ and 8^th^ week of starting the treatment regimens.

***Results:*** At the end of the 2^nd^ week we achieved cure rates of 72% and 64.3% for group 1 and group 2 respectively. At the end of the 4^th^ week the respective cure rates for group 1 and group 2 were 81.2% and 69%, and at the end of the 8^th^ week 70.8% of the patients in group 1 and 61.9% of the patients in group 2 were cured.

***Conclusion:*** The results of this study showed no significant statistical differences between the two groups in regard to cure and recurrence rates. But the numbers of cured patients were higher and recurrent cases were lower in group 1.

## INTRODUCTION

Pityriasis versicolor (tinea versicolor) is a common mild infection of the skin which is caused by the lipophilic yeast Malassezia furfur.^1^ Colonization of the skin by this organism more frequently occurs in areas with high sebaceous activity and the disease is more common during adolescence and young adulthood.^2^ This condition is common in tropics and sex distribution is equal.^3^ Clinically the disease is characterized by scaly hyperpigmented and hypopigmented lesions which can occur simultaneously in any patient.^4^

The lesions primarily appear on the upper trunk, but involvement of the neck, upper arms, abdomen, inguinal areas and less commonly axilla, popliteal fossa, lower limbs and genitalia can occur. Sometimes the lesions may become confluent and produce large patches. The lesions are often asymptomatic but sometimes pruritus may be present.^3^^,^^5^^,^^6^ Skin lesions of hypopigmented type may occasionally appear on the forehead of the children.^7^^,^^8^

Malassezia furfur is considered a component of cutaneous normal flora which under certain conditions transforms into its pathogenic mycelial form and produces skin lesions of pityriasis versicolor. Poor hygiene, chronic infections, hyperhidrosis, malnutrition, prolonged use of steroids or broad spectrum antibiotics, stress, pregnancy and genetic factors may contribute to the development of pityriasis versicolor.^3^

A variety of oral and topical antifungal medications are available for the treatment of pityriasis versicolor. Topical treatment with propylene glycol lotions, ciclopirox olamine, topical azole creams, antidandruff shampoos, Artemesia sieberi lotion and terbinafine cream and systemic therapy with imidazole antifungals and oral terbinafine have resulted in different cure and recurrence rates.^9^^,^^10^

Terbinafine is an allylamine medication with a broad spectrum antifungal activity. Inhibition of squalene epoxidase by allylamines results in sterol deficiency in the fungal cell wall and also intracellular accumulation of squalene and finally fungal cell death.^11^ In one in vitro study terbinafine showed a weak effect against Malassezia furfur.^12^ But another study revealed beneficial effect of terbinafine in the treatment of pityriasis versicolor.^13^

There have been a small number of studies on the effect of terbinafine in the treatment of pityriasis versicolor. Considering the inconsistent results of these studies, we decided to conduct this comparative study of terbinafine versus ketoconazole for the treatment of pityriasis versicolor. The aim of this study was to compare the clinical efficacy of terbinafine cream with that of ketoconazole cream in the treatment of pityriasis versicolor.

## METHODS

This randomized single blind clinical trial included 110 patients 14 years of age and older with clinical diagnosis of pityriasis versicolor. The diagnosis was confirmed by potassium hydroxide (KOH) microscopy. This study was approved by Ethical Review Committee of Kurdistan University of Medical Sciences and was in accordance with the principles of the Declaration of Helsinki.

According to the rule of nines the skin lesions must have involved 10% or less of the body surface area. Pregnant and breast-feeding women, patients who had used topical or systemic antifungal medications or steroids within 30 days before starting our study and patients with extensive skin lesions were excluded from the study. After obtaining written informed consent, the patients were assigned to two groups. Each group consisted of 55 patients. Group 1 used terbinafine hydrochloride 1% cream and group 2 patients were treated with ketoconazole 2% cream. Both groups used their topical medications twice daily for a period of two weeks.

Clinical and microscopic examinations were carried out at baseline and at the end of the 2^nd^, 4^th^ and 8^th^ weeks of starting the study. Clinical assessment was performed on the basis of scoring the severity of pruritus, erythema and scaling from zero to 3 (3=severe, 2=moderate, 1=mild 0=absent). Patients with negative mycological examination either with clearance of skin lesions or presence of mild residual disease were considered cured.^14^

Demographic data including age, gender, site of skin lesions, clinical signs and symptoms were recorded in a check-list for each of the patients at baseline and at the end of the 2^nd^, 4^th^, and 8^th^ weeks of starting the study. Data were collected and introduced into SPSS Win 16 software. Chi-square test and Fisher`s exact test were used for data analysis.

**Figure:**
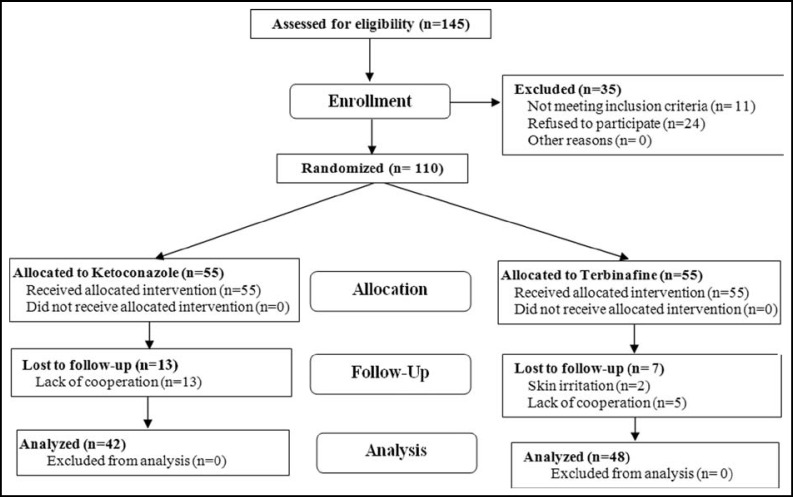


## RESULTS

This study included 110 patients. Only 90 patients (48 patients in group 1 and 42 patients in group 2) completed the study. There were no statistically significant differences between the two groups in regard to age, gender, occupation, location and distribution of the lesions (p>0.05). The mean ages of the patients in group 1 and group 2 were 27.25±8.46 and 26.26±8.6 years, respectively. In both groups most patients were between 21 and 30 years of age. 47.9% of the patients in group 1 and 61.9% of the patients in group 2 were high school and university students (P=0.64). The locations of the skin lesions were similar in both groups and included chest, back, abdomen and neck, in descending order. In some of the patients more than one location had been involved (Table-I).

**Table-I:** Demographic characteristics of the patientients in group 1 and group 2.

**Groups**	**Group 1 (Terbinafine)**	**Group 2 (ketoconazole)**	
**Variables**	**N(%)**	**N(%)**	
**Age **			
20 years and younger	13(27.1)	12(28.6)	0.4
21-30	18(37.5)	17(40.5)	
31-40	13(27.1)	11(26.2)	
41 and older	4(8.3)	2(4.8)	
**Gender **			
Male	28(66.7)	28(58.3)	0.41
Female	20(33.3)	14(41.7)	
**Occupation **			
High school and university students	23(47.9)	26(61.9)	0.64
Housewife	5(10.4)	4(9.5)	
Employee	12(25.0)	7(16.7)	
Other occupations	8(16.7)	5(11.9)	
**Location of skin lesions**			
Chest	30(58.8)	28(58.3)	0.97
Back	9(17.7)	8(7/16)	
Abdomen	7(13.7)	6(12.5)	
Neck	5(9.8)	6(12.5)	

**Table-II:** Comparison of the therapeutic effects of terbinafine cream with ketoconazole cream at the end of the 2^nd^, 4^th^ and 8^th^ week of study.

**Time**	**Cure**	**Group 1 (Terbinafine)**	**Group 2 (ketoconazole)**	**p**
		**N(%)**	**N(%)**	
End of the 2^nd^ week	Cured	35(72.1)	27(64.3)	0.38
	Not cured	13(27.9)	15(35.7)	
End of the 4^th^ week	Cured	39(81.2)	29(69.0)	0.8
	Not cured	9(18.8)	13(31.0)	
End of the 8^th^ week	Cured	34(70.8)	26(61.9)	0.37
	Not cured	14(29.2)	16(38.1)	

The respective cure rates for group 1 and group 2 were 72.1% and 64.3% at the end of the 2^nd^ week, 81.2% and 69% at the end of the 4^th^ week, and 70.8% and 61.9% at the end of the 8^th^ week which did not show significant statistical differences (Table-II). At the end of the 8^th^ week we found recurrence rates of 1.3% and 2.4% for group 1 and group 2 respectively which did not revealed any significant difference.

## DISCUSSION

In this study we found a higher cure rate in the patients of group 1, at the end of the 2^nd^ week of the treatment course. Also examination of the patients at each follow up visit revealed a higher rate of successful therapy in the patients who had used terbinafine which suggested that terbinafine, although not statistically significant, had been more effective than ketoconazole in the treatment of pityriasis versicolor.

Several studies have shown the effectiveness of topical ketoconazole and also its superiority to placebo, 2.5% selenium sulfide shampoo, 10% sulfur and 3% salicylic acid soap for the treatment of pityriasis versicolor.^15^^-^^19^ Bhogal and his colleagues concluded that oral fluconazole had been more effective than oral ketoconazole.^20^ In another study Rigopoulos showed that ketoconazole shampoo and flutrimozole shampoo had similar therapeutic effect for this fungal infection.^21^

In one study Faergemann concluded that terbinafine 1% gel was more effective than placebo, after a 7 day treatment course.^22^ Aste and his colleagues assessed the clinical efficacy and tolerability of terbinafine 1% cream versus bifonazole 1% cream in the patients with pityriasis versicolor and reported cure rates of 100% and 95% for the patients in terbinafine and bifonazole groups respectively. They also showed that terbinafine was a well tolerated medication with rapid action.^11^

In a placebo controlled study, Vermeer achieved clinical and mycological cure rates of 72% and 81% respectively, which is compatible with the results of our study.^23^

Chopra and his colleagues conducted a comparative clinical trial of topical terbinafine and ketoconazole and found a clinical and mycological clearance of 88% in ketoconazole group and 96% in terbinafine group.^24^ The cure rates in our study were less than those of Chopra`s study, but in both studies topical terbinafine was more effective than topical ketoconazole for the treatment of pityriasis versicolor.

## CONCLUSION

According to the results of this study terbinafine and ketoconazole had similar effects for the treatment of pityriasis versicolor. But use of terbinafine resulted in a higher rate of therapeutic success and a lower recurrence rate.
